# Synchronized bursts of productivity and success in individual careers

**DOI:** 10.1038/s41598-022-10837-1

**Published:** 2022-05-10

**Authors:** Sumit Kumar Ram, Shyam Nandan, Sami Boulebnane, Didier Sornette

**Affiliations:** 1 Department of Management, Technology and Economics, ETH Zürich, Scheuchzerstrasse 7, 8092 Zurich, Switzerland; 2 MIT Connection Science, Massachusetts Institute of Technology, Cambridge, USA; 3 Department of Earth Sciences, ETH Zürich, Zurich, Switzerland; 4 Department of Physics, ETH Zürich, Zurich, Switzerland; 5 Swiss Finance Institute c/o University of Geneva, Geneva, Switzerland; 6 Tokyo Tech World Research Hub Initiative, Institute of Innovative Research, Tokyo Institute of Technology, Tokyo, Japan; 7 Institute of Risk Analysis, Prediction and Management (Risks-X), Academy for Advanced Interdisciplinary Studies, Southern University of Science and Technology (SUSTech), Shenzhen, 518055 China

**Keywords:** Statistical physics, Nonlinear phenomena

## Abstract

Notwithstanding a significant understanding of epidemic processes in biological, social, financial, and geophysical systems, little is known about contagion behavior in individual productivity and success. We introduce an epidemic model to study the contagion of scholarly productivity and YouTube success. Our analysis reveals the existence of synchronized bursts in individual productivity and success, which are likely mediated by sustained flows of information within the networks.

## Introduction

Human lives are driven by their social networks^1^, whose structure determines what they do and achieve^2^. Thus, a better understanding of these networks is likely to help predict performance, productivity, and success of individuals^2–6^. While individuals are capable of driving their own productivity, their success is often dependent on perceptions by others within their networks^2^. The stochastic flow of information propagating through the networks could influence individual productivity and success trajectories^2^. The primary aim of this paper is to explore how this flow of information further influence the productivity and success of collectives? Here, we uncover the presence of strong social influence and of stochastic flows of information within social networks fueling an epidemic process, which could further lead to exuberant production and consumption of creative content. This leads to synchronized bursts of productivity and success within individual careers.

The ‘science of success’ has received a boost in recent years with the growing availability of large datasets describing individual’s careers from which much can be learned and importantly predicted. There is a growing body of literature that recognises the importance of various factors like, person’s reputation^2^, sustained productivity^7^, impact of recognition^8^, luck^9^, status effect^10^, competition^11^, etc. in individuals’ success and productivity patterns. There is also a large volume of published studies describing the role of networks in individuals’ productivity and success. For instance, Fraiberger et al.^2^ investigates, how a person’s reputation and networks of influence play a key role in determining access to resources and rewards in areas of human activity where performance is difficult to quantify in an objective fashion. Li et al.^12^ discusses the long-term impact of coauthorship with highly-cited scientists on the careers of junior researchers. They find that junior researchers who coauthored work with top scientists enjoyed a persistent competitive advantage throughout the rest of their careers. Azoulay el al.^10^ discusses the concept of a status effect, which is when actors are given differential recognition for their efforts depending on their location in a status ordering. They examined the impact of a major status-conferring prize on citations to articles published by the scientist before the prize was awarded. They found evidence of a post-appointment citation boost, but the effect was small and limited to a short window of time. Ma et al.^13^ discusses the link between mentorship and protégé success. The study finds that mentorship is associated with diverse forms of protégé success, significantly increasing protégé chances of producing celebrated research, being inducted into the National Academy of Science, and achieving superstardom. Paradoxically, protégé achieve their highest impact when they display intellectual independence from their mentors. protégé do their best work when they break from their mentor’s research topics and coauthor no more than a small portion of their overall research with their mentors.

A range of studies have tried to shed light on the importance of network effect derived from the affiliated institution on one’s success and productivity trajectory and vice versa. Keith et al.^14^ examines the relationship between prestige and scholarship in the discipline of sociology. They found that departmental prestige is primarily a function of faculty scholarship. In contrast, Burris^15^ argued that academic department prestige is not based on the scholarly productivity of the faculty and graduates, rather it is based on a department’s position within social networks of association and social exchange. Th other way around, Ponomariov et al.^16^ examines the effect of university research centers on the productivity and collaboration patterns of university faculty. They found that affiliation with a university research center can increase productivity and facilitate cross-disciplinary and cross-sector collaborations. This is further supported by Way et al.^17^, that looked at the correlation between productivity and prominence of faculty at prestigious universities and less prestigious universities. They found that the pattern of productivity and prominence was not due to selection effects (i.e. the competitive job market), but rather due to the work environments of the universities. Conversely, Horta et al.^18^ found negative productivity patterns because of academic inbreeding (The practice of having Ph.D. graduates employed by the university) on scholarly practices and achievement. Cruz et al.^19^ investigated the relationship between scientific performance and rewards, with a focus on the mediating effect of mobile versus stable career paths. The study found that inbred faculty do not get tenure with less scientific merits than PhDs from other institutions, and that non-mobile careers are a strong predictor of the timing of rewards in the form of early permanent positions.

Contrary to previously established understanding, that is there is no statistically predictable patterns in individuals’ success trajectory, recent studies demonstrated the presence of statistically robust patterns. Liu et al.^6^ and Ram et al.^20^ uncovered the presence of “hot streaks”—persistent periods during which someone’s performance is significantly better than usual—among artists, film directors, and scientists, and sports performers and argued that this phenomenon is not due to random chance. Williams et al.^7^ showed how success in the acting industry is not always determined by high impact, but rather by sustained productivity. They found that the dynamics of job assignment is well described by a “rich-get-richer” mechanism and that productivity tends to be higher towards the beginning of a career. Janosov et al.^9^ showed how luck can be considered a crucial ingredient to achieve impact in all creative domains, and provided new insights on the role of randomness in impact in creative careers. They showed that luck consistently affects career impact across all considered sectors, and improved our understanding in pinpointing the key elements in driving success. Li et al.^8^ and Wang et al.^21^ discussed the importance of certain events in one’s success and productivity trajectory. Li et al.^8^ studied the Nobel Prize winners in physics, chemistry, and physiology or medicine. The study found that, although Nobel laureates were energetic producers from the outset, their careers before winning the prize follow relatively similar patterns to those of ordinary scientists. The study also found that there are notable variations along their careers, often associated with the Nobel Prize. Wang et al.^21^ discusses the long-term effects of setbacks experienced by junior scientists. The study found that an early-career setback has powerful, opposing effects—it significantly increases attrition, predicting more than a 10% chance of disappearing permanently from the NIH system, yet despite this, individuals with near misses systematically outperform those with narrow wins in the longer run. This suggests that the experience of a setback can cause a performance improvement among those who persevere.

A number of studies also found an association between factors like competition^11^, mobility^22^ and parenthood^23^ that particularly influence productivity. Certo et al.^11^ reported how competition has influenced scholarly productivity in the field of management. They found that the number of scholars publishing papers each year in top-tier management outlets increased significantly over time. However, the majority of scholars required more than 5 (or 10) years to publish five (or ten) top-tier articles. The study also found that increased competition to publish articles in top-tier journals has affected the scholarly productivity of both micro and macro researchers, with the negative influence on productivity being more pronounced for macro scholars. Dietz et al.^22^ examined the correlation between job changes and productivity among scientists and engineers working at university research centers in the United States. They found that job changes throughout a scientist or engineer’s career can provide access to new social networks and scientific and technical human capital, which leads to increased productivity. Morgan et al.^23^ looked at the impact of parenthood on scholarship. They found that parenthood explains most of the gender productivity gap by lowering the average short-term productivity of mothers. However, the size of the productivity penalty for mothers appears to have shrunk over time.

The existing literature on science of success is extensive and focuses particularly on the productivity and success patterns of individuals. However, studies remain limited with respect to dealing with the success and productivity cycles driven by exogenous sources of influence. There is also a relatively small body of literature that is concerned with how and what factors affect the success and productivity of the collectives. The aim of this article is therefore to understand the effect of these exogenous sources/shocks on success and productivity trajectories of the collectives that get subjected to these shocks. The rest of the article is structured as follows. “Methods” describes the dataset and the methods that have been used in the study. “Results” summarizes the key observations. “Discussion” presents an epidemic model to explain and validate the results. We conclude the results and provide the limitations of the study in “Conclusion”.

## Methods

### Data

We develop a temporal topic-based extraction to mine two networks. First, we consider a network of scholars working and publishing in the same scientific discipline, here Signal Processing as an example, within a given timeframe. Second, we consider a network of content creators on YouTube creating content on a particular topic: cryptocurrency as an example. We use state-of-the-art Natural Language Processing (NLP) and data aggregation techniques to prepare the datasets for our study. We analyze and extract data from the Microsoft academic graph^24^ dataset containing $\sim 213$ million scholarly articles. Similarly, we analyze $\sim 100$ million YouTube videos (see Supplementary Material for data preparation).

**Figure 1:**
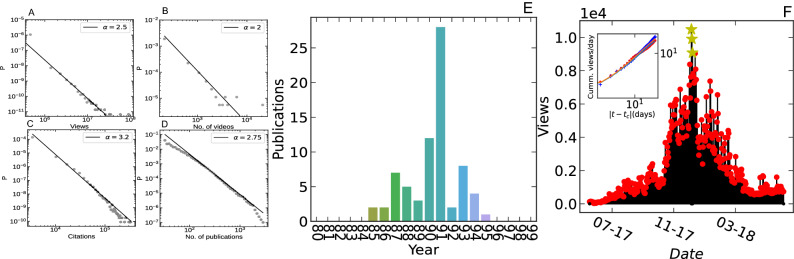
(**A**) Scaling behavior of total view counts per video in YouTube dataset. (**B**) Distribution of total number of videos per YouTube content creator. (**C**) Scaling of total citations per author in Microsoft Academic Graph database. (**D**) Scaling behavior (with logarithmic binning) of number of publications per author. (**E**) An illustration of the typical response found in selected channel’s view time series on YouTube. (Inset) The cumulative precursory (“foreshock”) and relaxation (“aftershock”) on a log-log scale, revealing the power-law behavior that lasts over months. (**F**) Annual productivity (no. of publications/year) within a sample scholarly career.

Figure 1 illustrates a few stylized facts about the two datasets. We observe several similarities in terms of their statistical properties. Firstly, the distribution of view counts and the total number of citations for YouTube content creators and authors, which indicate the success of individuals, follow a power-law scaling behavior (Fig. 1A,C). Secondly, the distribution of total number of videos created by YouTube content creators and total number of publications by authors, which point to the productivity of individuals, follow power-law scaling behavior (Fig. 1B,D).

### Productivity in scientific career

Understanding individual scientific productivity can help develop policies that improve each scientist’s ability to succeed and enhance the prospects of Science as a whole^4^. Several factors can influence scientific productivity, like age^3^, collaboration network^4^, scientific discipline^5^. Knowledge creation is a complex process^4^, and scholars from the same discipline often imitate, collaborate, and compete to develop understanding. This creates herding between the scholars who tend to follow the same research direction. Here we quantify the influence of the herding behavior on individual productivity.

We first identify the scholars who published articles on a scientific particular discipline (see Supplementary Material for data preparation). We then count the total number of publications each year by a scholar on a particular scientific discipline to construct the productivity time series. We call it annual scientific productivity. For this study, we illustrate the yearly scientific productivity of the authors publishing on the topic of signal processing as example. Panel E in Fig. 1 presents a scientific productivity trajectory for a randomly selected scholar.

We then find the peak of productivity $(t_c)$, that is the year of the highest number of publications. To avoid the domination of highly productive careers in our statistical analysis, we normalize the productivity path of each scholar by its maximum productivity value. We then perform a peak-centered average (*A*(*t*)) of the annual productivity across all the authors. (*A*(*t*)) and $(t_c)$ are the key parameters that we study through out our analysis.

### Success in YouTube

Effort, skill, or inherent excellence, as well as luck^25^, can drive success. While it is difficult to quantify the effort and its role^25^, the attention flowing through social networks is one of the drivers of success^2–6^. The position of the performer in social networks^2^ and the perception of the artistic work within peers^2–6^ can influence and facilitate success. Thus, the performers constantly imitate, collaborate, and compete with each other to attract the attention of the spectators, which leads to similar content creation. Unable to quantify the quality of the content, spectators start to imitate other’s choices and start herding.

We consider the YouTube platform^26^ to quantify the evolution of success that results from the herding behavior of spectators. The key success element in YouTube is the total number of views of the artist’s content, as this decides his/her earning. We consider the total number of weekly views (from all contents) as the success metric for a YouTube channel. Here, we illustrate the success of Cryptocurrency content creators as an example. Panel F in Fig. 1 presents a YouTube success trajectory for a randomly selected content creator.

We then find the peak of success $(t_c)$, that is the week of the highest number of views. Again, we normalize the success path of each channel by its maximum value of weekly views, to avoid the domination of highly successful channels in our statistical analysis. We then perform a peak-centered average (*A*(*t*)) of the weekly view counts across all the channels.

### Ethical approval

All methods were carried out in accordance with relevant guidelines and regulations.

## Results

### Scaling in individual productivity and success

**Figure 2:**
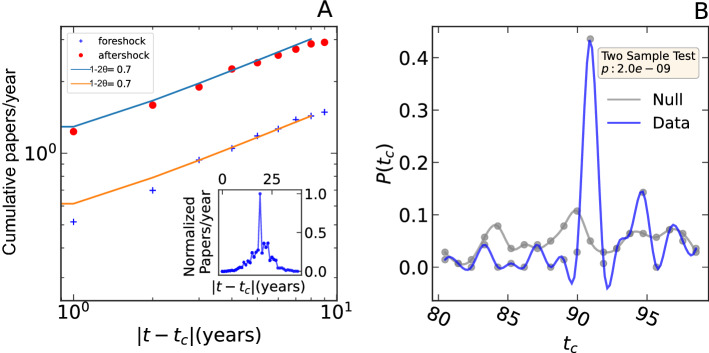
Synchronized endogeneity in scientific productivity: (**A**) cumulative precursory (“foreshock”) and relaxation (“aftershock”) of renormalized scholarly annual productivity of 140 scholarly careers. The exponents of scaling laws are 0.7. The inset figure shows the peak-centered average annual scholarly productivity. (**B**) The probability distribution of time of maximum annual productivity in scholarly careers (in blue). The distribution of time of maximum productivity in randomly distributed publications within the careers (in gray). The p value of two-sample Kolmogorov–Smirnov test result is given in the yellow box.

**Figure 3:**
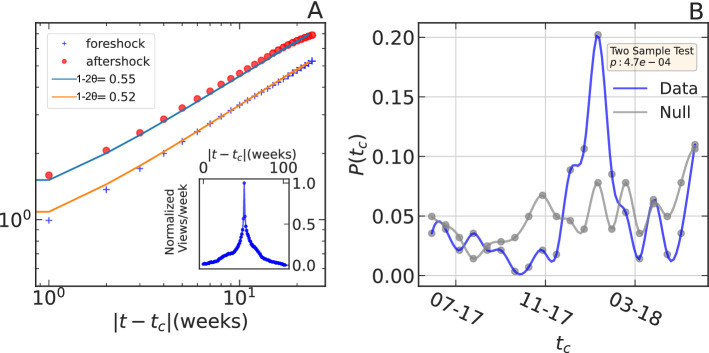
Synchronized endogeneity in YouTube success: (**A**) cumulative precursory (“foreshock”) and relaxation (“aftershock”) of weekly view counts of the 399 YouTube content creators (see text). The scaling laws are with exponent $\sim 0.5$. The inset figure shows the peak-centered average weekly views. (**B**) The probability distribution of the time of the highest weekly views for the YouTube channels. The blue line is obtained from the data, and the gray line is for the null distribution constructed by randomly shuffling the daily weekly counts. The p value of the two-sample Kolmogorov–Smirnov test result is given in the yellow box.

The annual productivity of an author is the sum of all publications on Signal Processing in a given year. The peak of productivity $(t_c)$ is the year of the highest number of publications. *A*(*t*) is the peak-centered average of the annual productivity across all the authors. In the inset of Fig. 2A, one can observe that the precursory and post-peak dynamics are governed by similar growth and decay patterns. For the YouTube data, the peak of success $(t_c)$ is the week of the highest number of YouTube views. As for scholarly productivity, we observe and quantify the burst of success in YouTube. The inset in Fig. 3A shows identical growth patterns in pre-peak and post-peak dynamics. The identical scaling behavior is further validated in the main figure. We plot the cumulative sum of the renormalized average weekly view counts for pre-peak (foreshock) and post-peak (aftershock) dynamics. For both cases, the exponent quantifying the scaling behavior is $\sim 0.5$. We randomly shuffle both datasets 100 times to prepare the null hypothesis test to check that the results are not spurious and would not be present by chance. We thus determine the significance of the obtained exponent values^27^. We perform the Wilcoxon signed-rank test to determine the statistical significance. We observe that both the pre-peak and post-peak exponent obtained from data is significantly different than the null $(p < 10^{-6})$.

**Figure 4:**
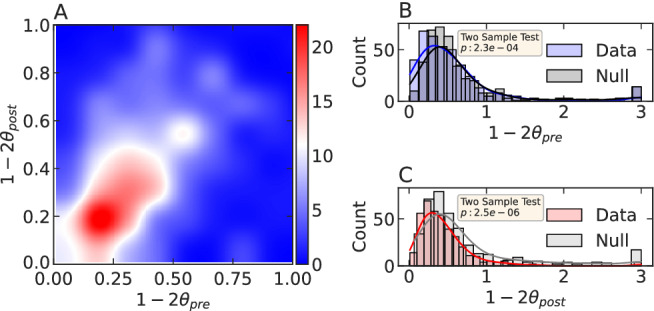
Joint and marginal distribution of pre-peak and post-peak relaxation exponents in YouTube success: we calibrate the individual daily channel view time series to find the dependence of pre- and post-peak exponents. (**A**) Joint distribution of pre-peak and post-peak exponents. The highest density of points cluster near 0.5. The fuzzy C-mean clustering gives the centroid of the distribution to be $\sim (0.5, 0.5)$. (**B**) Marginal distribution of pre-peak exponents obtained from the data and the pre-peak exponents obtained from randomly shuffled null data. The two-sample Kolmogorov–Smirnov test suggests that the distribution of the exponents obtained from data is significantly different than that of the exponents in null. (**C**) same as (**B**), except the exponent values represent the post-peak exponents.

Using an iterated least-squares fit technique^26^, we calibrate the evolution of daily views. We observe unimodal distributions of relaxation exponents for both cases centered around 0.5 (see Fig. 4B,C). The joint distribution of pre-peak and post-peak exponents cluster around $(\sim 0.5, \sim 0.5)$. Further, these exponents are significantly different from those obtained from randomly shuffled null datasets.

### Synchronization of productivity and success

We note down the time when the number of publications and number of views were maximum within each career. Figure 2B shows the distribution of these times $(t_c)$ (in year), when the scholars published their maximum number of papers. Surprisingly, we witness a peak within the distribution around the year 1991. We evaluate the significance of the observed peak by comparing it with a null distribution. We randomly redistribute the publications within each career, determine the year of maximum publications, and obtain the corresponding distribution in the gray line. A two-sample Kolmogorov–Smirnov (KS) test shows that the peak of the distribution of the non-shuffled data is significant. We repeat the above analysis with the YouTube data to better understand the bursts of success. Figure 3B shows the distribution of dates $(t_c)$ of the highest YouTube views within each success trajectory. Similar to Fig. 2B, we observe a peculiar peak around January 2018. The small p value from the KS test confirms the significance of the peak. The rejection of the null hypothesis for both datasets supports the hypothesis that endogenous growth of productivity and success can synchronize, likely due to herding and synchronized exuberant behaviors. Thus, in addition to the evidence for endogenous success (corroborated by the symmetric nature of the productivity peaks), there is an exogenous component at the level of individuals, which is endogenous to their global network via feedback herding loops.

## Discussion

The endogenously fueled spontaneous interplay between a continuous stochastic flow of small external impulses and the amplifying impact of the epidemic cascade of influences results in bursts of productivity and success^28^. Thus, the bursts of productivity and success in individual careers can be effectively modeled with a simple epidemic branching process^26^. According to this model, the productivity or success at a time $t_i$ triggers the productivity or success at a future time $t_i$. The influence of past on future productivity or success decays with a memory kernel $\phi \left( t,t_i\right)$. With the assumption that the distribution of interevent time of human activity follows a power-law behavior^28^, this memory kernel $\phi \left( t,t_i\right)$ can be expressed by^26,29–31^,

1 $$\begin{aligned} \phi (t,t_i) \sim \frac{1}{(t-t_i)^{1+\theta }} \quad with\quad 0<\theta <1 \end{aligned}$$

The intensity $\lambda (t)$ of the point process describing the arrival of papers or videos results from a combination of exogenous $(\mu (t))$ and endogenous sources. Because of endogeneity, each past success or productivity can become the cause of future success or productivity according to the term $\sum _{t_i\le t}\phi \left( t,t_i\right)$ in the expression (2).

2 $$\begin{aligned} \lambda (t) \sim \mu (t)+\sum _{t_{i} \le t} \phi \left( t, t_{i}\right) \end{aligned}$$

In the absence of major external influence, the bare kernel $\phi (t,t_i)$ of this epidemic process in Eq. (1) is renormalized into the dressed kernel *A*(*t*) according to Eq. (3)^26,29–31^. The renormalized activities in *A*(*t*) decay symmetrically around the peak $t_c$ according to

3 $$\begin{aligned} A(t)=\frac{1}{\left| t-t_{c}\right| ^{1-2 \theta }} \end{aligned}$$

Figures 2A and 3A present the time dependence of *A*(*t*) (in Eq. 3) of the epidemic process (in Eq. 2). The endogenous propagation of influence is confirmed by the similar exponent values of the cumulative pre-peak (foreshock) and post-peak (aftershock) temporal growth patterns (see Figs. 2A and 3A). The results further provide an estimate of the exponent for the power-law influence kernel in an individual’s productivity and success patterns. We estimate that, on average, there is an influence of productivity on future productivity decays as $\phi \left( t\right) \sim \frac{1}{t^{1.15}}$, i.e., $\theta \sim 0.2$ as defined in expression (1) and the past views trigger the cascades of future views with a memory kernel $\phi \left( t\right) \sim \frac{1}{t^{1.25}}$, i.e., $\theta \sim 0.2$ (using Eqs. 1, 3). Additionally, we show that the model accurately predicts the post-peak relaxation rate based on pre-peak dynamics. By studying the pre-peak and post-peak relaxation exponents of all the YouTube channels, we show that the joint distribution of pre-peak and post-peak exponents cluster around $(\sim 0.5, \sim 0.5)$ (see Fig. 4A), as predicted by the theory (Eq. 3) of endogenous peaks^26,29–31^.

## Conclusion

The main question explored in this study was concerned with the global influence of exogenous sources/shocks in individuals’ productivity and success trajectories. Our investigation showed the emergence of synchronized bursts of individual productivity and success, which revealed the existence of herding between performers and spectators. By stacking the peaks (after normalization), we showed that the productivity and success trajectories are symmetric before and after the peak. Furthermore, they can be represented by a power-law with exponent 1–2$\theta$, where 1–2$\theta$ takes approximately the same value before and after the peak. We found 1–2$\theta \sim 0.7$ for the scholarly productivity and $\sim 0.55$ for the YouTube success. These results translate to an endogenously fueled epidemic branching process within each career, with a slowly decaying influence kernel $(\phi \left( t\right) \sim \frac{1}{t^{\ 1+\theta }})$, with $\theta =$ 0.15–0.25. The findings of this investigation complement those of earlier studies^26,29–31^, with a relatively smaller value of $\theta$ (i.e., $\theta =$ 0.3–0.4), signifying the slower decay of the influence in the present case.

Interestingly, we observe the peak of productivity around 1991 in most scholarly careers concerned with signal processing and the peak of success around Jan 2018 in most of the YouTube channels. We can trace the synchronized herding in scholarly productivity back to a wave of interest in wavelets during the 1990s (“Wavelet revolution” during the 1990s). The herding in YouTube success occurs one month after the peak in December 2017 of the cryptocurrency bubble (2017 Cryptocurrency mania). Thus, we demonstrate endogenously fueled herding effects in each case, where the scholars and the YouTubers, and the respective communities were most active during the two times of focus. The presence of exuberance within the performers (in the case of scholarly productivity) and exuberance within the spectators (in the case of YouTube success) led to a synchronization of the bursts of productivity and success. One of the more significant findings revealed from this study is the emergence of endogenously fueled exuberant behavior within the networks caused by the shocks which are exogenous in origin. These findings have significant implications for the understanding of global productivity and success of individuals’ working in a particular direction.

Although the study has successfully demonstrated the impact of exogenous shocks on productivity and success trajectories of individuals with two examples, it has certain limitations in terms of the scalability of the applied methodology. We curated the YouTube dataset with customized vocabulary to filter the desired content^32^ (see Supplementary Material). This approach cannot be scaled to filter and investigate arbitrary topics. Furthermore, even though we started our investigation with relatively large datasets, after conditioning on several dimensions (i.e., topic based filtering, filtering on minimum career size, etc.), we ended up having a relatively small number of careers. The above shortcomings can be addressed by applying video analysis tools^33,34^ to cluster the videos based on their contents. Notwithstanding these limitations, the study suggests the strong systemic influence of network synchronisation in individuals’ productivity and success.

## Supplementary Information

Supplementary Information.

## Data Availability

All study data are included in the article.
