# Supplementary material for: Synchronized bursts of productivity and success in individual careers
Source: Sci Rep. 2022 May 10;12:7637. doi: 10.1038/s41598-022-10837-1 (PMC9091239; doi:10.1038/s41598-022-10837-1)
Supplement: Supplementary file 1 — Supplementary Information. [file 41598_2022_10837_MOESM1_ESM.pdf]

Supplementary Materials: Synchronized bursts of productivity and success in individual careers

Sumit Kumar Ram[1,2*], Shyam Nandan[†3], Sami Boulebnane[†4], and Didier Sornette[1,3-7*]

[1]Department of Management, Technology and Economics, ETH Zürich, Scheuchzerstrasse 7, 8092, Zürich, Switzerland
[2]MIT Connection Science, Massachusetts Institute of Technology, Cambridge, USA
[3]Department of Earth Sciences, ETH Zürich, Zürich, Switzerland
[4]Department of Physics, ETH Zürich, Zürich, Switzerland
[5]Swiss Finance Institute c/o University of Geneva, Geneva, Switzerland
[6]Tokyo Tech World Research Hub Initiative, Institute of Innovative Research, Tokyo Institute of Technology, Tokyo, Japan
[7]Institute of Risk Analysis, Prediction and Management (Risks-X), Academy for Advanced Interdisciplinary Studies, Southern University of Science and Technology (SUSTech), Shenzhen, 518055, China
[†]*Contributed equally to this work*
[*]*sram@ethz.ch,dsornette@ethz.ch*

January 2022

1 Supplementary Materials

1.1 Materials and Methods

1.1.1 Preparation of citation data

Our study uses a database containing $\sim$ 256 million scholars, who have published $\sim$ 213 million articles on $\sim$ 53 thousand research topics. We mine the database to extract all the articles on the topic of Signal Processing that have been published between 01/01/1971 and 01/01/2000. We find a total of $\sim$ 36 thousand publications within this duration. We further match these publications with their authors to reconstruct the careers. To accurately identify the productivity peak in each career, we consider only the authors with at least 20 scientific articles.

1.1.2 Preparation of YouTube data

We develop a technique to classify channels that create content on a particular topic on YouTube. We analyze the voice inside the videos with state-of-the-art Natural Language Processing (NLP) techniques. We categorize the videos and subsequently the channels. We extracted the information about the evolution of view counts, likes, shares, subscribes for the channel creating similar content at a particular time point.

1.1.3 Quasi-Monte Carlo search for finding related YouTube channels

YouTube provides a list of suggested channels that might be interesting for the viewers. We use the above feature to sample the list of channels, which might be creating similar content. We manually select few YouTube channels that create content related to Cryptocurrency. We use them as initial seeds to query YouTube and sample the list of channels using a Quasi-Monte Carlo search algorithm. First, we find the list of suggested channels for a given channel. Further, we proceed with the search by finding the list of suggested channels for the suggested channels and so on. We redo the query with many initial conditions for sampling. We sample a list of $\sim$ 1 million YouTube channels that have created 100 million videos. However, this doesn't ensure that the contents in the channels are on the same topic. Hence we move to the next step for further processing.

1.1.4 Analyzing the contents of the videos

We systematically analyze the content of each video from the YouTube channels. We use Google's speech-to-text synthesizer to extract the content of the videos in text format. We then use this text for analyzing and matching the voices to classify videos based on the content. We analyze the texts using a customized NLP algorithm that we develop to categorize the videos. For this, we first construct a vocabulary containing thousands of words that can be used to recognize the conversations related to Cryptocurrency. With the help of this vocabulary and the Gestalt matching [1], we classified the cryptocurrency videos.

1.1.5 Reconstructing the YouTube channel's career

After classifying the videos, we map back the videos with the channels and categorize the channels. We extract the evolution of the daily view count for the videos. We sum the daily view counts from all the videos to find the daily view counts for the YouTube channel.

References

[1] John W Ratcliff and David E Metzener. Pattern-matching-the gestalt approach. *Dr Dobbs Journal*, 13(7):46, 1988.
